# Development and validation of a prognostic model to predict the prognosis of patients with colorectal gastrointestinal stromal tumor: A large international population-based cohort study

**DOI:** 10.3389/fonc.2022.1004662

**Published:** 2022-11-02

**Authors:** Yiding Li, Yujie Zhang, Yang Fu, Wanli Yang, Xiaoqian Wang, Lili Duan, Liaoran Niu, Junfeng Chen, Wei Zhou, Jinqiang Liu, Jing Wang, Daiming Fan, Liu Hong

**Affiliations:** ^1^ State key Laboratory of Cancer Biology and National Clinical Research Center for Digestive Diseases, Xijing Hospital of Digestive Diseases, Fourth Military Medical University, Xi’an, China; ^2^ Department of Histology and Embryology, School of Basic Medicine, Xi’an Medical University, Xi’an, China; ^3^ School of Basic Medical Sciences, Fourth Military Medical University, Xi’an, China; ^4^ Department of Immunology, Fourth Military Medical University, Xi’an, China

**Keywords:** colorectal gastrointestinal stromal tumors, nomogram, prognostic factors, survival rate, SEER database

## Abstract

**Background:**

Colorectal gastrointestinal stromal tumors (GISTs), mesenchymal malignancy, only accounts for about 6% of GISTs, but prognosis is generally poor. Given the rarity of colorectal GISTs, the prognostic values of clinicopathological features in the patients remain unclear. Nomograms can provide a visual interface to help calculate the predicted probability of a patient meeting a specific clinical endpoint and communicate it to the patient.

**Methods:**

We included a total of 448 patients with colorectal GISTs diagnosed between 2000 and 2019 from the Surveillance, Epidemiology, and End Results (SEER) database. For nomogram construction and validation, patients in the SEER database were divided randomly into the training cohort and internal validation cohort at a ratio of 7:3, while 44 patients with colorectal GISTs from our hospital patient data set between 2010 to 2016 served as the external validation cohort. The OS curves were drawn using the Kaplan–Meier method and assessed using the log-rank test. And, Fine and Gray’s competing-risks regression models were conducted to assess CSS. We performed univariate and multivariate analyses to select prognostic factors for survival time and constructed a predictive nomogram based on the results of the multivariate analysis.

**Results:**

Through univariate and multivariate analyses, it is found that age, primary site, SEER stage, surgery, and tumor size constitute significant risk factors for OS, and age, primary site, histological grade, SEER stage, American Joint Committee for Cancer (AJCC) stage, surgery, and tumor size constitute risk factors for CSS. We found that the nomogram provided a good assessment of OS and CSS at 1-, 3- and 5- year in patients with colorectal GISTs. The calibration plots for the training, internal validation and external validation cohorts at 1-, 3- and 5- year OS and CSS indicated that the predicted survival rates closely correspond to the actual survival rates.

**Conclusion:**

We constructed and validated an unprecedented nomogram to predict OS and CSS in patients with colorectal GISTs. The nomogram had the potential as a clinically predictive tool for colorectal GISTs prognosis, and can be used as a potential, objective and additional tool for clinicians in predicting the prognosis of colorectal GISTs patients worldwide. Clinicians could wield the nomogram to accurately evaluate patients’ OS and CSS, identify high-risk patients, and provide a baseline to optimize treatment plans.

## Introduction

Although gastrointestinal stromal tumors (GISTs) are rare, they are among the most common mesenchymal malignancy in the whole digestive tract, with an annual worldwide incidence of 11 to 19.6 per million (1, 2), originating from the interstitial cells of Cajal (3, 4). Overall GISTs therefore affect a significant proportion of cancer patients. GISTs can occur in any part of the alimentary tract, most commonly in the stomach (55.6%), small intestine (31.8%), and less frequently in the colon and rectum (6%) (5). Researchers found that colorectal GISTs, with the worst overall survival and the poorest prognosis than the other GISTs, which is one of the most challenging problems faced by surgeons (6, 7). Meanwhile, most of the studies have shown that colonic GISTs presents the overall worst prognosis, greater metastatic potential, and higher relapse rate compared to rectal GISTs (6, 8), while a few have also suggested that patients with tumors located in different colonic locations and the rectum have similar prognosis (9). Complete surgical resection is currently considered the standard treatment for patients with resectable GISTs with the goal of curing. Unfortunately, GISTs recurrence, even after the entire resection of the tumor and negative margins, is still frequently observed in clinical settings (10, 11). In recent decades, there has been a great interest in determining the genetic characteristics of these neoplasms, and it was identified and determined that GISTs are characteristically driven by activating mutations of KIT in approximately 85–90% of cases (12–14). Recent studies have shown that introducing effective tyrosine kinase inhibitors (TKIs), particularly imatinib, have been investigated as potential treatments for GISTs with inoperable or metastatic disease (15, 16). In 2002, TKIs was first approved by the FDA for the treatment of patients with metastatic GISTs, which improved median overall survival (OS) from less than 1 year to 5 years (17, 18). The drug was later approved for adjuvant use in resectable GIST, which improved OS from 35 to 83% (18, 19).

Given the scarcity of colorectal GISTs, the meager amount of research, and the inadequacy of studies, the prognostic values of clinicopathological features and treatments in patients remain uncertain. Therefore, recurrent or metastatic risk stratification of GISTs has been attempted worldwide, which is mainly based on tumor size, mitotic activity and location (8, 10). In addition, the modified National Institutes of Health consensus criteria (NIH criteria of 2008) and the WHO Classification of Tumors of the Digestive System consensus criteria (WHO criteria of 2013) are the most commonly used staging systems for prognosis (20, 21). However, neither of these classifications can answer the question about survival rates, especially the survival time for each individual. As it happened, nomogram is a simple pictorial representation of a statistical prediction model to assist in easy and rapid predictions and clinical decision-making in clinical practice. It generates a precise prediction based on the assessment of important factors and provides accurate, individualized risk predictions for each individual for estimating the conditional risk of disease outcomes.

In this study, we aim to analyze and compare the prognostic features of colorectal GISTs using a relatively prodigious amount of cases obtained from the Surveillance, Epidemiology, and End Results (SEER) database and to develop a delicate nomogram based on significant prognostic factors to predict 1-, 3-, and 5-year overall survival (OS) and cancer specific survival (CSS). Further, we verified the prognostic value of the prediction model using an external validation set from our hospital database.

## Methods

### Data source and population selection

This study used data from two sources - the SEER database and the Xijing Hospital database. The first source was from the SEER database provided by the National Cancer Institute’s SEER*Stat software version 8.4.0.1 (https://seer.cancer.gov/datasoftware/). The screening of patients with colorectal GISTs was as follows: 1) patients came from the database of “SEER Research Plus Data, 17 Registries, Nov 2021 Sub (2000-2019)”; 2) the International Classification of Diseases for Oncology (ICD-O) site codes caecum (C18.0), ascending colon (C18.2), hepatic flexure of the colon (C18.3), transverse colon (C18.4), splenic flexure of colon (C18.5), descending colon (C18.6), sigmoid colon (C18.7), rectosigmoid junction (C19.9) and rectum (C20.9) were used to identify patients; and 3) according to “Histologic Type ICDO-3,” the following pathological types were included in this study: GISTs (8936). We only included patients positively diagnosed with histology tests and excluded the patients with incomplete survival information. Since the SEER database contains no identifiers and were publicly available, the approval of the institutional review board was not required. The second source comprised of colorectal GISTs patients who were diagnosed and received treatment at Xijing Hospital from 2010 to 2016. The included patients from our center were approved by the Ethical Committee of Xijing Hospital, with orally informed consents. The study was performed in accordance with the ethical standards laid down in the 1964 Declaration of Helsinki and its later amendments.

We extracted demographic information (age, sex, race), clinicopathological characteristics (primary site, histological grade (grade), tumor size, SEER stage, American Joint Committee for Cancer (AJCC) stage, AJCC T stage, AJCC N stage, AJCC M stage), primary treatment modality (surgery, chemotherapy and radiotherapy), survival time, vital status, and cause-specific death classification at the last follow-up from the chosen cases. The study used OS and CSS as the primary end point, and results are presented as hazard ratios (HRs) with 95% confidence intervals (CIs). OS is defined as time to death from any cause, and CSS was defined as the time to death from colorectal GISTs. X-tile software version 3.6.1 (Yale University School of Medicine, US) was used to analyze the optimal cutoff point of age and tumor size (22). The X-tile plot cut-off for tumor size was 65 mm and the cut-off for age was 65 and 80. Additionally, race was categorized as white, black, or other (American Indian/Alaska Native, Asian or Pacific Islander, Hispanic). Primary site was categorized as right-sided colon (caecum, ascending colon, hepatic flexure of the colon, or transverse colon), left-sided colon (splenic flexure of colon, descending colon, or sigmoid colon), and rectum (rectosigmoid junction or rectum).

### Missing data

Missing values for candidate predictors were handled with multiple imputation (R package MICE). The imputation model included all candidate predictors and outcomes (time to event). The resulting 10 complete datasets were separately analyzed and the results combined with Rubin’s rules to produce overall estimates and confidence intervals.

### Statistical analysis

Statistical analyses were conducted using SPSS 20.0 (SPSS, Inc., Chicago, IL) and R software (version 4.1.2). Descriptive statistics are presented as frequencies (*n*) and percentages (*%*). To estimate cancer survival probabilities, we considered death due to the diagnosed cancer as the event of interest and death due to other causes as the censoring event. We used Fine and Gray’s competing risk analysis (23) to estimate the cumulative incidence function (CIF) to explore each single variable incidence of each competing event. Moreover, we used the proportional sub-distribution hazard model to identify the significant variables associated with CSS and constructed the competing risk nomogram based on these factors to assess the association between predictor variables and the outcomes. OS curves were calculated with the Kaplan-Meier method and were analyzed with the log-rank test. Univariate and multivariate survival analyses were performed using the Cox proportional hazard regression model. All variables were included, and the variables that showed a statistically significant effect (P < 0.05) in the univariable analysis were entered in the multivariable Cox proportional hazards model. A multivariable fractional polynomial (MFP) approach is commonly adopted in medical research (24–26) to determine the importance of variables and their functional forms for model development. The MFP approach was carried out in R using the package “mfp”(Ambler and Benner) (27). Then, the nomogram for OS was constructed based on the results of the multivariable analysis. Variables selected for inclusion were carefully chosen to ensure parsimony of the final models. The proportional hazard assumption was tested using the Schoenfeld residuals test (28) for each measure, and no violations of this assumption were detected. Moreover, the variance inflation factor (VIF) was estimated to check for multicollinearity (29).

For nomogram construction and validation, patients in the SEER database were randomly grouped into training cohort and internal validation cohort according to a ratio of 7:3, and harnessed our hospital patient data set as the external validation cohort. All incorporating prognostic variables from the training cohort were included to create the nomogram that predicted the probability of a patient’s survival rate at 1, 3, or 5 years. Each subtype of the factors on the nomogram corresponds to a point on the “Point” scale. The points for each variable are summed together to generate a total-point score. The total-point scores projected on the bottom scales indicate the probabilities of 1-, 3-, and 5-year OS or CSS. Validation of each nomogram consisted of three procedures in training, internal validation, and external validation cohorts. First, the area under the curve (AUC) of the receiver operating characteristic (ROC) was used to evaluate the discrimination performance of the nomogram, TNM staging system, and histological grade, which ranges from 0.5 (no discrimination) to 1.0 (perfect discrimination, equivalent to the standard). AUC measures the discrimination ability of the different staging systems to stratify patients with different outcomes: higher the AUC, better the model is about a patient’ prognosis. Second, the calibration plot was performed using a bootstrap method with 1,000 resamples to compare the agreement between actual observed and predicted survival rates. Intuitively, the closer the simple regression line between the actual and predicted survival rates is to the diagonal, the closer the predicted survival rates are to the actual survival rates. All statistical tests used a significance level of 5% in a two-tailed test.

## Results

### Clinical characteristics

After excluding patients with missing follow-up data, a total of 448 patients with colorectal GISTs diagnosed between 2000 and 2019 were selected from the SEER database and assigned to the training cohort (n = 313) and the internal validation cohort (n = 135). For the whole cohort, the median follow-up time was 80.50 months (interquartile range, 26.25–132.50 months). Number of deaths from any cause was 246 and number of deaths from colorectal GISTs was 146. Based on the same inclusion and exclusion criteria as the SEER cohort, 44 patients with colorectal GISTs were included in the external validation cohort from the Xijing Hospital. Of the total SEER group, the demographic and clinical characteristics did not differ between the training and internal validation cohorts. **Table 1** summarizes the general demographic and clinicopathological characteristics of the patients in the SEER database. Patients diagnosed with right-sided colonic GISTs (*n* = 69), left-sided colonic GISTs (*n* = 60), rectum GISTs (*n* = 184), were included in the study for comparisons. The above data suggest that patients with rectum GISTs had much more cases than patients with right-sided colonic GISTs and left-sided colonic GISTs. In addition, we found that the proportion of male patients was higher than that of female patients with colorectal GISTs. Roughly three-quarters of the patients (*n* = 336 [75.00%]) underwent surgery. Less than half of patients (*n* = 193 [43.08%]) received definitive chemotherapy, whereas only 5.58% patients received radiation. Tumor size was less than 65 mm in 51.79% of patients, and greater than or equal to 65 mm in 48.21% of patients. In addition, we found that patients with rectum GISTs were younger and received less surgical resection than those with colonic GISTs. Patients with colonic GISTs had a higher proportion of distant disease, and had significantly more proportion of tumors with size >65 mm, especially left-sided colonic GISTs. These results are summarized in **Supplementary Table 1**. Demographic and clinical characteristics of the patients chosen from Xijing Hospital are listed in **Supplementary Table 2**. Patients diagnosed with right-sided colonic GISTs (*n* = 9), left-sided colonic GISTs (*n* = 8), rectum GISTs (*n* = 27), were included in the study for comparisons. Concerning the treatment strategy, 68.18% patients with colorectal GISTs underwent surgery, while the remain 31.82% of patients with colorectal GISTs did not. Tumor size was less than 65 mm in 52.27% of patients, and greater than or equal to 65 mm in 47.73% of patients. These results were consistent with results from the SEER database.

**Table 1:** Demographics and Clinicopathologic Characterisitcs of Patients with Colorectal GISTs.

Cetegory	Trainning cohort (*n* = 313)		Internal validation cohort (*n* = 135)		Total cohort (*n* = 448)		*P* ^§^	External validation cohort (*n* = 44)		P^¶^
	*n*	*%*	*n*	*%*	*n*	*%*		*n*	*%*	
**Age**										
<65	163	52.08%	68	50.37%	231	51.56%	0.861	22	50.00%	0.893
65-79	110	35.14%	51	37.78%	161	35.94%	17	38.64%
≥80	40	12.78%	16	11.85%	56	12.50%	5	11.36%
**Sex**										
Female	130	41.53%	55	40.74%	185	41.29%	0.876	12	27.27%	0.048
Male	183	58.47%	80	59.26%	263	58.71%	32	72.73%
**Race**										
White	213	68.05%	83	61.48%	296	66.07%	0.203	–	–	–
Black	48	15.34%	30	22.22%	78	17.41%	–	–
Other^†^	52	16.61%	22	16.30%	74	16.52%	–	–
**Primary Site**										
Right-sided	69	22.04%	27	20.00%	96	21.43%	0.727	4	9.09%	0.028
Left-sided	60	19.17%	30	22.22%	90	20.09%	5	11.36%
Rectum	184	58.79%	78	57.78%	262	58.48%	35	79.55%
**Histological Grade** ^‡^										
I/II	146	46.65%	77	57.04%	223	49.78%	0.055	21	47.73%	0.893
III/IV	167	53.35%	58	42.96%	225	50.22%	23	52.27%
**SEER Stage**										
Localized	173	55.27%	71	52.59%	244	54.46%	0.849	28	63.64%	0.522
Regional	79	25.24%	35	25.93%	114	25.45%	8	18.18%
Distant	61	19.49%	29	21.48%	90	20.09%	8	18.18%
**AJCC Stage**										
I/II	124	39.62%	57	42.22%	181	40.40%	0.606	21	47.73%	0.305
III/IV	189	60.38%	78	57.78%	267	59.60%	23	52.27%
**AJCC T Stage**										
T1-2	113	36.10%	49	36.30%	162	36.16%	0.969	–	–	–
T3-4	200	63.90%	86	63.70%	286	63.84%	–	–
**AJCC N Stage**										
N0	296	94.57%	130	96.30%	426	95.09%	0.437	–	–	–
N1	17	5.43%	5	3.70%	22	4.91%	–	–
**AJCC M Stage**										
M0	272	86.90%	117	86.67%	389	86.83%	0.946	–	–	–
M1	41	13.10%	18	13.33%	59	13.17%	–	–
**Surgery**										
No	78	24.92%	34	25.19%	112	25.00%	0.953	12	27.27%	0.736
Yes	235	75.08%	101	74.81%	336	75.00%	32	72.73%
**Chemotherapy recode**										
No	178	56.87%	77	57.04%	255	56.92%	0.974	–	–	–
Yes	135	43.13%	58	42.96%	193	43.08%	–	–
**Radiation recode**										
No	294	93.93%	129	95.56%	423	94.42%	0.492	–	–	–
Yes	19	6.07%	6	4.44%	25	5.58%	–	–
**Tumor size**										
<65 mm	157	50.16%	75	55.56%	232	51.79%	0.294	25	56.82%	0.408
≥65 mm	156	49.84%	60	44.44%	216	48.21%	19	43.18%

† American Indian/AK Native, Asian/Pacific Islander, unknown; ‡ Grade I: well differentiated; Grade II: moderately differentiated; Grade III: poorly differentiated; Grade IV: undifferentiated; § Comparisons between trainning cohort and internal validation cohort were performed using Pearson's chi- square test and p values less than 0.05 were considered significant. ¶ Comparisons between trainning cohort and external validation cohort were performed using Pearson's chi- square test and p values less than 0.05 were considered significant. mm, millimeter; Colorectal GISTs, Colorectal gastrointestinal stromal tumors.

### Survival analysis

**Supplementary Figure 1** shows the Kaplan–Meier survival curves of OS for patients by age, sex, race, primary site, grade, SEER Stage, AJCC Stage, AJCC T stage, AJCC N stage, AJCC M stage, surgical options, chemotherapy recode, radiation recode, and tumor size. Kaplan–Meier survival curves of OS showed that patients with older age, higher grade, higher AJCC stage, AJCC T3-4 stage, AJCC M1 stage, enlarged tumor, and increased severity of the SEER stage had a relatively poor OS, while patients who underwent surgery had a beneficial effect on OS compared with those who did not. As for primary site, patients with rectum GISTs had a significantly longer OS compared to patients with the right-sided colonic GISTs and left-sided colonic GISTs, while the OS of the patients with the right-sided colonic GISTs and left-sided colonic GISTs did not show a significant difference.

**Supplementary Figure 2** shows the cumulative incidence function curves of CSS for patients by age, sex, race, primary site, grade, SEER Stage, AJCC Stage, AJCC T stage, AJCC N stage, AJCC M stage, surgical options, chemotherapy recode, radiation recode, and tumor size. The cumulative incidence function curves of CSS presented that patients with older age, higher grade, higher AJCC stage, AJCC T3-4 stage, AJCC M1 stage, enlarged tumor, and increased severity of the SEER stage had a relatively poor CSS, while patients who underwent surgery had a beneficial effect on CSS compared with no surgery. As for primary site, patients with rectum GISTs had a significantly longer CSS compared to patients with the right-sided colonic GISTs and left-sided colonic GISTs, while the CSS of the patients with the right-sided colonic GISTs and left-sided colonic GISTs did not show a significant difference.

**Table 2** summarizes an exploratory univariate analysis examining impact of multiple candidate factors on OS and CSS. Factors including age, primary site, grade, SEER stage, AJCC stage, AJCC T stage, AJCC M stage, tumor size, and surgery were significantly related to OS and CSS through univariate analysis. The multivariate analysis includes all significant variables found in the univariate analysis (**Table 3**). After adjustment for possible confounders, we considered that age, primary site, SEER stage, surgery, and tumor size constitute significant risk factors for OS, and age, primary site, histological grade, SEER stage, AJCC stage, surgery, and tumor size constitute risk factors for CSS. Moreover, no significant multicollinearity was found among the variables. The VIF scores for the variables are all relatively low (VIF < 2).

**Table 2:** Univariate analysis for Overall survival and Cancer-specific survival in training cohort.

Cetegory	*n*	Overall survival *P* (Log-rank test)	Cancer-specific survival *P* (Fine and Gray’s test)
Age			
<65	163	Ref.	Ref.
65-79	110	<0.001	<0.001
≥80	40	<0.001	<0.001
Sex			
Female	130	Ref.	Ref.
Male	183	0.830	0.124
Race			
White	213	Ref.	Ref.
Black	48	0.168	0.165
Other^†^	52	0.121	0.424
Primary Site			
Right-sided	69	Ref.	Ref.
Left-sided	60	0.925	0.174
Rectum	184	0.029	0.004
Histological Grade^‡^			
I/II	146	Ref.	Ref.
III/IV	167	<0.001	<0.001
Seer Stage			
Localized	173	Ref.	Ref.
Regional	79	<0.001	<0.001
Distant	61	<0.001	<0.001
AJCC Stage			
I/II	124	Ref.	Ref.
III/IV	189	<0.001	<0.001
AJCC T Stage			
T1-2	113	Ref.	Ref.
T3-4	200	0.006	<0.001
AJCC N Stage			
N0	296	Ref.	Ref.
N1	17	0.710	0.065
AJCC M Stage			
M0	272	Ref.	Ref.
M1	41	0.007	0.027
Surgery			
No	78	Ref.	Ref.
Yes	235	<0.001	0.023
Chemotherapy recode			
No	178	Ref.	Ref.
Yes	135	0.765	0.894
Radiation recode			
No	294	Ref.	Ref.
Yes	19	0.159	0.121
Tumor size			
<65 mm	157	Ref.	Ref.
≥65 mm	156	<0.001	<0.001

^†^ American Indian/AK Native, Asian/Pacific Islander, unknown.

^‡^ Grade I: well differentiated; Grade II: moderately differentiated; Grade III: poorly differentiated; Grade IV: undifferentiated;

The P value in the column of univariate analysis means that the variable was selected in the next multivariate analysis.

mm, millimeter; Ref., referent.

**Table 3:** Multivariate analysis for Overall survival and Cancer-specific survival in training cohort.

Cetegory	Overall survival		Cancer-specific survival	
	*HR (95% CI)*	*P*	*HR (95% CI)*	*P*
Age				
<65	Ref.	—	Ref.	—
65-79	3.22 (2.24, 4.61)	<0.001	2.56 (1.76, 3.72)	<0.001
≥80	7.79 (4.85, 12.52)	<0.001	5.77 (3.44, 9.67)	<0.001
Primary Site				
Right-sided	Ref.	—	Ref.	—
Left-sided	0.72 (0.45, 1.13)	0.150	0.50 (0.31, 0.81)	0.005
Rectum	0.59 (0.39, 0.91)	0.016	0.54 (0.36, 0.82)	0.004
Histological Grade^†^				
I/II	Ref.	—	Ref.	—
III/IV	1.32 (0.89, 1.94)	0.162	1.71 (1.14, 2.54)	0.009
SEER Stage				
Localized	Ref.	—	Ref.	—
Regional	1.31 (0.90, 1.92)	0.159	1.41 (0.93, 2.13)	0.108
Distant	2.81 (1.57, 5.06)	<0.001	2.53 (1.42, 4.52)	0.002
AJCC Stage				
I/II	Ref.	—	Ref.	—
III/IV	1.48 (0.94, 2.31)	0.088	1.95 (1.18, 3.23)	0.009
AJCC T Stage				
T1-2	Ref.	—	Ref.	—
T3-4	0.67 (0.38, 1.17)	0.155	0.64 (0.34, 1.23)	0.184
AJCC M Stage				
M0	Ref.	—	Ref.	—
M1	0.74 (0.41, 1.33)	0.311	0.71 (0.39, 1.27)	0.248
Surgery				
No	Ref.	—	Ref.	—
Yes	0.48 (0.27, 0.85)	0.012	0.46 (0.22, 0.95)	0.035
Tumor size				
<65 mm	Ref.	—	Ref.	—
≥65 mm	2.16 (1.27, 3.67)	0.005	3.07 (1.70, 5.52)	<0.001

^†^ Grade I: well differentiated; Grade II: moderately differentiated; Grade III: poorly differentiated; Grade IV: undifferentiated;

mm, millimeter; Ref., referent.

### Nomogram construction and validation

**Figure 1:**
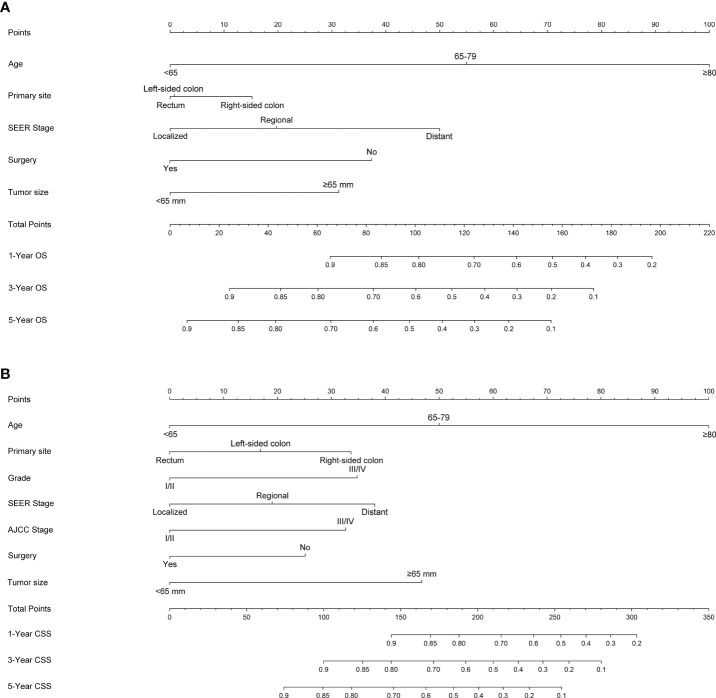
Nomogram predicting 1-, 3- and 5-year **(A)** OS, **(B)** and CSS of patients with Colorectal GISTs. Summarizing the scores of each variable together and the total points projected on the bottom scales indicate the probabilities of 1-, 3- and 5-year OS and CSS. OS, overall survival; CSS, cancer-specific survival; Colorectal GISTs, Colorectal gastrointestinal stromal tumors.

**Figure 2:**
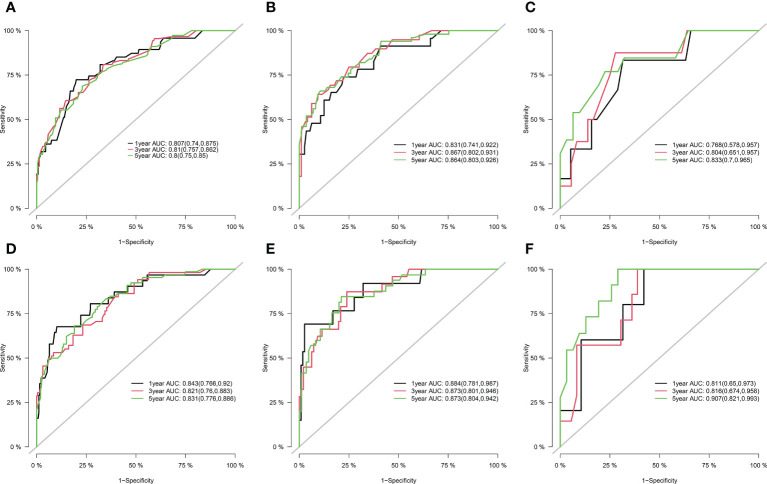
ROCs curve for the nomograms in predicting prognosis in patients with Colorectal GISTs. **(A)** ROC of 1-, 3- and 5-year OS in the training cohort; **(B)** ROC of 1-, 3- and 5-year OS in the internal validation cohort; **(C)** ROC of 1-, 3- and 5-year OS in the external validation cohort; **(D)** ROC of 1-, 3- and 5-year CSS in the training cohort; **(E)** ROC of 1-, 3- and 5-year CSS in the internal validation cohort; **(F)** ROC of 1-, 3- and 5-year CSS in the external validation cohort. ROC, receiver operating characteristic; OS, overall survival; CSS, cancer-specific survival; Colorectal GISTs, Colorectal gastrointestinal stromal tumors.

**Figure 3:**
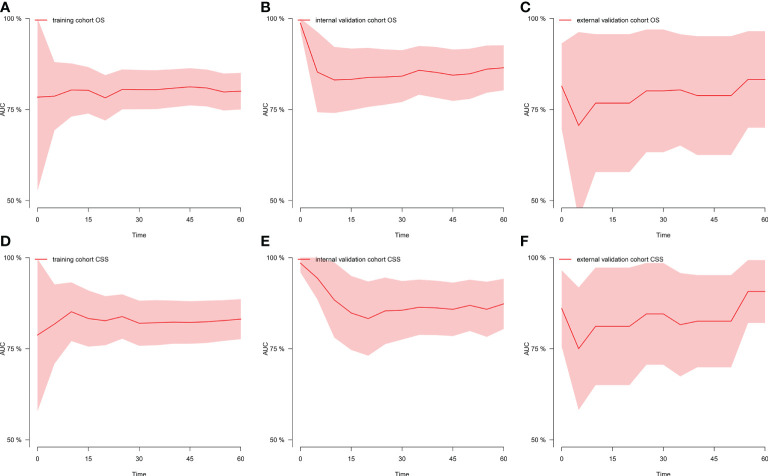
Time-dependent AUC at each time point. **(A)** Time-dependent AUC of OS in the training cohort; **(B)** Time-dependent AUC of OS in the internal validation cohort; **(C)** Time-dependent AUC of OS in the external validation cohort; **(D)** Time-dependent AUC of CSS in the training cohort; **(E)** Time-dependent AUC of CSS in the internal validation cohort; **(F)** Time-dependent AUC of CSS in the external validation cohort. AUC, the area under the curve value of the receiver operating characteristic; OS, overall survival; CSS, cancer-specific survival.

**Figure 4:**
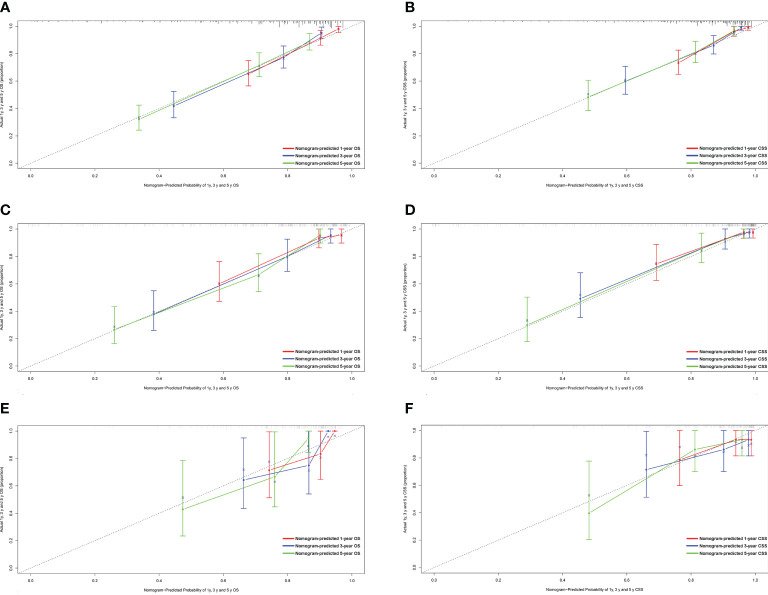
Calibration plots of the nomogram for 1-, 3- and 5-year OS and CSS prediction. **(A)** Calibration curves of 1-, 3- and 5-year OS in the training cohort; **(B)** Calibration curves of 1-, 3- and 5-year CSS in the training cohort; **(C)** Calibration curves of 1-, 3- and 5-year OS in the internal validation cohort; **(D)** Calibration curves of 1-, 3- and 5-year CSS in the internal validation cohort; **(E)** Calibration curves of 1-, 3- and 5-year OS in the external validation cohort; **(F)** Calibration curves of 1-, 3- and 5-year CSS in the external validation cohort. OS, overall survival; CSS, cancer-specific survival.

**Figure 1** shows the prognostic nomogram of OS and CSS at 1, 3 and 5 years. Our nomograms show good identification and predictability. The predictive performance of the nomogram for 1-, 3-, and 5- year OS and CSS in the training, internal validation, and external validation cohorts was evaluated by the ROC curve. We found that the nomogram provided a good assessment of OS and CSS at 1, 3, and 5 years in patients with colorectal GISTs [1-year OS: (training cohort: AUC = 0.807 (95% CI, 0.740, 0.875); internal validation cohort: AUC = 0.831 (95% CI, 0.741, 0.922); external validation cohort: AUC = 0.768 (95% CI, 0.578, 0.957)]; 3 year OS: [training cohort: AUC = 0.810 (95% CI, 0.757, 0.862); internal validation cohort: AUC = 0.867 (95% CI, 0.802, 0.931); external validation cohort: AUC = 0.804 (95% CI, 0.651, 0.957)]; 5 year OS: [training cohort: AUC = 0.800 (95% CI, 0.750, 0.850); internal validation cohort: AUC = 0.864 (95% CI, 0.803, 0.926); external validation cohort: AUC = 0.833 (95% CI, 0.700, 0.965)]; 1 year CSS: [training cohort: AUC = 0.843 (95% CI, 0.766, 0.920); internal validation cohort: AUC = 0.884 (95% CI, 0.781, 0.987); external validation cohort: AUC = 0.811 (95% CI, 0.650, 0.973)]; 3 year CSS: [training cohort: AUC = 0.821 (95% CI, 0.760, 0.883); internal validation cohort: AUC = 0.873 (95% CI, 0.801, 0.946); external validation cohort: AUC = 0.816 (95% CI, 0.674, 0.958)]; 5 year CSS: [training cohort: AUC = 0.831 (95% CI, 0.776, 0.886); internal validation cohort: AUC = 0.873 (95% CI, 0.804, 0.942); external validation cohort: AUC = 0.907 (95% CI, 0.821, 0.993)]. The results are illustrated in **Figure 2** and **Table 4**. Compared with the nomogram, the AUC of the nomogram was higher than that of TNM stage [1 year OS: AUC = 0.676 (95% CI, 0.598, 0.753); 3 year OS: AUC = 0.682 (95% CI, 0.620, 0.744); 5 year OS: AUC = 0.719 (95% CI, 0.663, 0.774); 1 year CSS: AUC = 0.742 (95% CI, 0.666, 0.818); 3 year CSS: AUC = 0.747 (95% CI, 0.680, 0.814); 5 year CSS: AUC = 0.770 (95% CI, 0.712, 0.829)] and histological grade[1 year OS: AUC = 0.637 (95% CI, 0.569, 0.704); 3 year OS: AUC = 0.630 (95% CI, 0.573, 0.687); 5 year OS: AUC = 0.640 (95% CI, 0.585, 0.694); 1 year CSS: AUC = 0.672 (95% CI, 0.600, 0.744); 3 year CSS: AUC = 0.662 (95% CI, 0.597, 0.727); 5 year CSS: AUC = 0.652 (95% CI, 0.589, 0.716)]. The results are illustrated in **Supplementary Figure 3**. In **Figure 3**, the time-dependent AUC at each time point revealed that the nomogram had a higher AUC at all time points. **Figure 4** shows the calibration plots for the training, internal validation, and external validation cohorts at 1-, 3-, and 5- year OS and CSS. The results indicated that the predicted survival rates of 1, 3, and 5 years closely correspond to the actual survival rates.

**Table 4:** AUC for the Nomogram in patients with Colorectal GISTs.

Survival		Training cohort	Internal validation cohort	External validation cohort
Overall survival	at 1-year	0.807 (*95% CI*, 0.740, 0.875)	0.831 (*95% CI*, 0.741, 0.922)	0.768 (*95% CI*, 0.578, 0.957)
	at 3-year	0.810 (*95% CI*, 0.757, 0.862)	0.867 (*95% CI*, 0.802, 0.931)	0.804 (*95% CI*, 0.651, 0.957)
	at 5-year	0.800 (*95% CI*, 0.750, 0.850)	0.864 (*95% CI*, 0.803, 0.926)	0.833 (*95% CI*, 0.700, 0.965)
Cancer -specific surviva	at 1-year	0.843 (*95% CI*, 0.766, 0.920)	0.884 (*95% CI*, 0.781, 0.987)	0.811 (*95% CI*, 0.650, 0.973)
	at 3-year	0.821 (*95% CI*, 0.760, 0.883)	0.873 (*95% CI*, 0.801, 0.946)	0.816 (*95% CI*, 0.674, 0.958)
	at 5-year	0.831 (*95% CI*, 0.776, 0.886)	0.873 (*95% CI*, 0.804, 0.942)	0.907 (*95% CI*, 0.821, 0.993)

Colorectal GISTs, Colorectal gastrointestinal stromal tumors; CI, confidence interval; AUC, the area under the curve value of the receiver operating characteristic.

## Discussion

Colorectal GISTs, mesenchymal malignancy, only accounts for about 6% of GISTs (5), but prognosis is generally poor (6a; 7). Although previous studies indicate that tumor size, mitotic index, surgery, AJCC stage can be powerful prognostic factors of survival and oncological events (8, 10, 30–35), it remains ambiguous whether clinicopathological features have prognostic value in patients with colorectal GISTs because of the rarity of this disease. Meanwhile, colorectal GISTs remain a predicament for clinicians and scientists to predict clinical behavior. Previous studies have reported the prognostic factors affecting prognosis in the patients with colorectal GISTs, but the majority of the published literature concerning the characteristics, incidence, survival, and treatment strategies of colorectal GISTs has been case reports or small case series (7, 36–38). Although these findings advanced our understanding of colorectal GISTs, they may be particularly susceptible to patient selection bias and institutional bias, and there is still a voracious need for more information on this uncommon malignant tumor. The competitiveness of the present population-based study of prognosis in the patients with colorectal GISTs include its large size with 448 participants at baseline with follow-up data and generalizability beyond a few institutions. Our work had a relatively prodigious number of cases to analyze and compare the prognostic features of colorectal GISTs and developed a nomogram to effectively predict 1-, 3-, and 5-year OS and CSS based on significant prognostic factors.

In this study, epidemiological and clinicopathologic characteristics were analyzed for patients with colorectal GISTs, and the related risk factors for patients’ prognosis were determined. Our present study identified AJCC stage, the most basic and widely used cancer staging system, as an independent prognostic predictor of CSS but not OS in patients with colorectal GISTs. This finding is in agreement with previous investigations showing that the higher AJCC stage represents a worse prognosis in the patients with small intestinal, duodenal, stomach GISTs (33, 39, 40), while no studies exist on the clinical prognosis importance of AJCC stage in colorectal GISTs. To our knowledge, our study act as a pioneer of demonstrating the prognostic importance of AJCC stage in patients with colorectal GISTs. Furthermore, our analysis identified histologic grade, an indicator of tumor aggressiveness, as a predictor of survival in patients with colorectal GISTs. This finding is consistent with previous studies showing that improved survival is directly associated with better histologic grade (41–44). Moreover, previous studies have demonstrated that complete surgical excision represents the gold standard for localized GISTs and another momentous prognostic factor (7, 45–47). We also found that patients who underwent resection had a significantly better prognosis than those who did not undergo surgical resection. Additionally, we found that patients with distant SEER staging had poorer OS and CSS than patients with localized and regional SEER staging. As suggested by other authors, patients with tumor invasion into adjacent structures may be more likely to develop residual microscopic or macroscopic disease after resection (48, 49). Furthermore, we found that tumor size is an independent risk factor for survival in patients with colorectal GISTs. As the other authors suggested, larger tumor size was associated with poor prognosis. However, they used the 5-cm threshold recommended by the AJCC staging system (6, 38), whereas the thresholds (6.5-cm) we used were derived from an analysis based on a large database using X-tile software version 3.6.1 (Yale University School of Medicine, US) (22). Several studies have revealed that the AJCC T-classification system should be interpreted with caution because it still has limited predictive value for the prognosis of multiple tumors (50, 51). Therefore, it was necessary to investigate the threshold of tumor size for each type of tumor in greater detail. The implications of primary site on outcome of patients with colorectal GISTs remains controversial in previous studies. Most of the studies have shown that colonic GISTs presents the overall worst prognosis, greater metastatic potential, and higher relapse rate compared to rectal GISTs (6, 8), while a few have also suggested that patients with tumors located in different colonic locations and the rectum have similar prognosis (9). In this study, we proposed that primary site was also an important indicator of prognosis. Patients with the rectum GISTs had a significantly longer OS compared to patients with the right-sided colonic GISTs and left-sided colonic GISTs, while the OS of the patients with the right-sided colonic GISTs and left-sided colonic GISTs did not show a significant difference. Additionally, patients with the rectum and left-sided colonic GISTs had a significantly longer CSS compared to patients with the right-sided colonic GISTs.

Although multifactorial analyses confirmed risk factors associated with patient prognosis, these variables did not allow for accurate and differentiated prediction of colorectal GISTs, especially for estimating survival rates for each individual. Therefore, specifically designed prognostic prediction models is of considerable importance in answering this question. The nomogram generates accurate predictions based on an assessment of important factors and provides pinpoint and personalized risk predictions for each individual to estimate the conditional risk of disease outcomes. And, we found no other literature had reported nomograms to accurately estimate the prognosis of patients with colorectal GISTs. Thus, studies with larger sample size involving multiple centers should be conducted to construct a nomogram to predict the prognosis of patients with colorectal GISTs. Our study at least partially fills this gap by creating a nomogram model to establish the OS and CSS of colorectal GISTs on the basis of a large database. We constructed and validated a nomogram predicting 1-year, 3- and 5-year OS and CSS in patients with colorectal GISTs. For nomogram construction and validation, patients in the SEER database were randomly grouped into a training cohort and internal validation cohort according to a ratio of 7:3, and utilized our hospital patient data set as the external validation cohort. Through univariate and multivariate analyses, we considered that age, primary site, SEER stage, surgery, and tumor size constitute significant risk factors for OS, and age, primary site, histological grade, SEER stage, AJCC stage, surgery, and tumor size constitute risk factors for CSS. We found that the nomogram provides a exceptional assessment of OS and CSS in patients with colorectal GISTs and AUCs for the model were high in all time points. In addition, the calibration plots stated clearly that the predicted survival of 1, 3, and 5 years had an important bearing on the actual survival, suggesting that the predictive performance of the nomogram was superb. With simple training, healthcare professionals, patients, and the public can quickly grasp the nomogram to assess the individualized risk predictions for each individual. Using the nomogram, it can be seen that an individual aged 70 (53 points for OS; 50 points for CSS), left-sided colon GISTs (2 points for OS; 18 points for CSS), grade III (34 points for CSS), regional SEER stage (20 points for OS; 19 points for CSS), and AJCC I stage (0 points for CSS), who has been treated with surgery (0 points for OS; 0 points for CSS) and, tumor size = 70mm (30 points for OS; 48 points for CSS) has a total point score of 105 for OS and 169 for CSS. This equates to 1-, 3-, and 5-year OS of 0.78, 0.56, and 0.43, and 1-, 3-, and 5year CSS of 0.85, 0.69, and 0.58, respectively.

Our study has several limitations that should be considered while interpreting our results. First, it is a retrospective analysis using a public database and, in this case, the avoidance of selection bias is difficult. Second, our nomogram provided individual predictions of OS for patients with five clinicopathological factors and CSS for patients with seven clinicopathological factors, lacking other additional variables which was reported to be significantly prognostic factor for the prognosis of patients with GISTs, such as mitotic rate, tumor rupture, Ki67 index (52–54). However, the SEER database does not contain these variables and future studies will need to incorporate them further into the analysis. Third, we were unable to obtain information on the use of TKIs and the duration of treatment. This lack of data on target therapy may affect the reported survival data. Fourth, the size of the external validation cohort was relatively scant and the patients were from a single center. Therefore, further studies on the prognostic impact of these factors worth strenuous digging in order to provide guidance for the treatment of colorectal GISTs.

## Conclusion

In conclusion, age, primary site, SEER stage, surgery, and tumor size constitute significant risk factors for OS of patients with colorectal GISTs, and age, primary site, histological grade, SEER stage, AJCC stage, surgery, and tumor size constitute risk factors for CSS. We constructed and validated a nomogram to predict OS and CSS in patients with colorectal GISTs. The nomogram had the latent capacity as a clinically predictive tool for colorectal GISTs prognosis, and can be used as a potential, objective and additional tool for clinicians in predicting the prognosis of colorectal GISTs patients worldwide. Clinicians can employ the nomogram to accurately evaluate patients’ OS and CSS, identify high-risk patients, and provide a baseline to optimize treatment plans.

## Data availability statement

The original contributions presented in the study are included in the article/**Supplementary Material**. Further inquiries can be directed to the corresponding author.

## Author contributions

YL and YZ participated in the design of this study and wrote the manuscript. LH and DF conceived the original idea, supervised the overall direction and planning of the project. YF, WY, XW, LD, LN, JC, WZ, JL and JW contributed to the acquisition of the data, analysis, and interpretation of the data. All authors contributed to the article and approved the submitted version.

## Funding

This project was supported by Project supported by the National Natural Science Foundation of China (No. 82073210), The grant of Shaanxi Province (No. 2019ZDLSF01-02-01) and Xjijng Zhutui Project. The funding bodies played no role in the design of the study and collection, analysis, and interpretation of data and in writing the manuscript.

## Conflict of interest

The authors declare that the research was conducted in the absence of any commercial or financial relationships that could be construed as a potential conflict of interest.

## Publisher’s note

All claims expressed in this article are solely those of the authors and do not necessarily represent those of their affiliated organizations, or those of the publisher, the editors and the reviewers. Any product that may be evaluated in this article, or claim that may be made by its manufacturer, is not guaranteed or endorsed by the publisher.
