# Supplementary material for: Development and validation of a prognostic model to predict the prognosis of patients with colorectal gastrointestinal stromal tumor: A large international population-based cohort study
Source: Front Oncol. 2022 Nov 2;12:1004662. doi: 10.3389/fonc.2022.1004662 (PMC9666406; doi:10.3389/fonc.2022.1004662)
Supplement: Supplementary file 1 [file Table_1.docx]

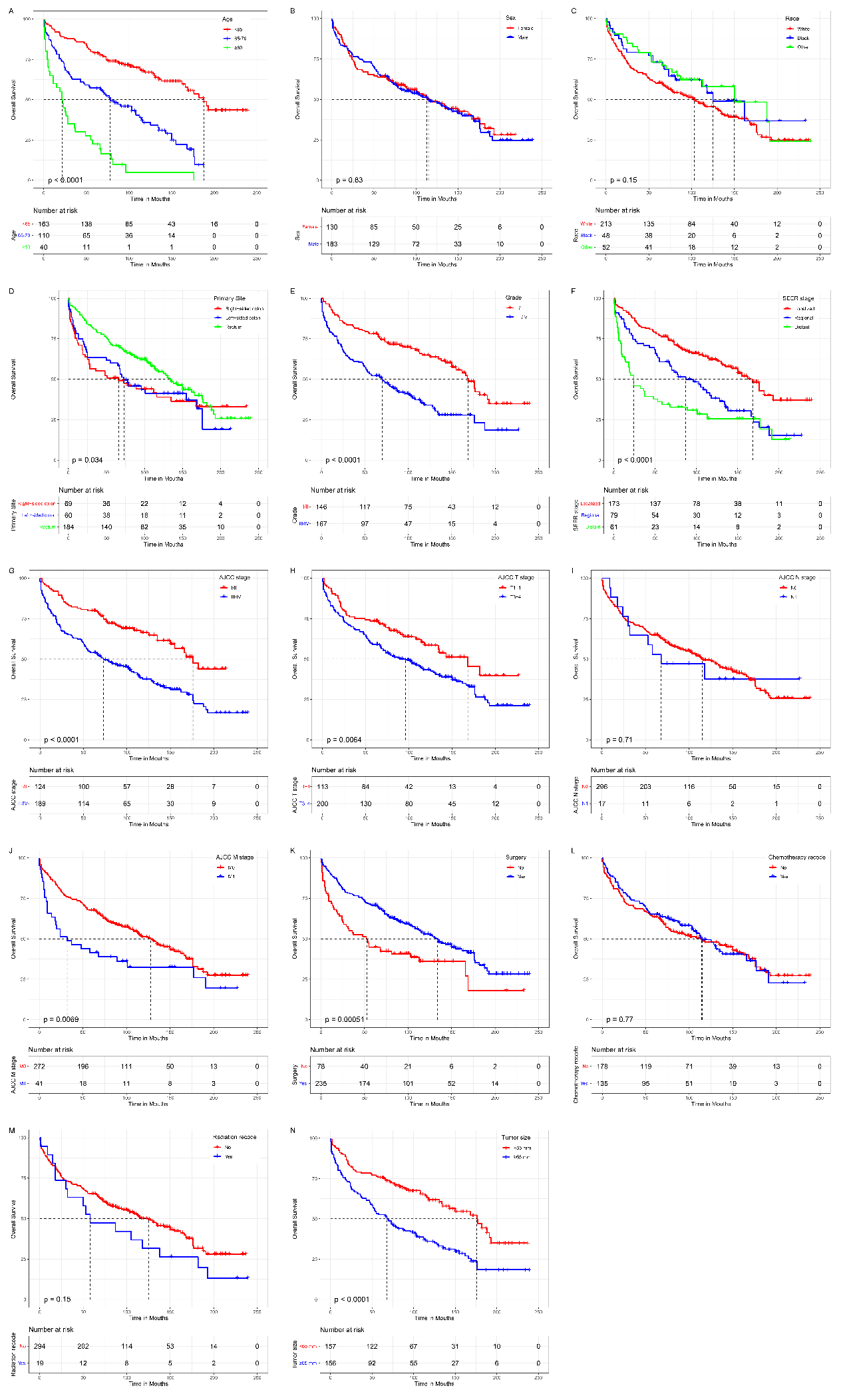


**Supplementary Figure 1** Kaplan–Meier curves of OS according to (A) Age, (B) Sex, (C) Race, (D) Primary site, (E) Grade, (F) SEER stage, (G) AJCC stage, (H) AJCC T stage, (I) AJCC N stage, (G) AJCC M stage, (K) Surgery, (L) Chemotherapy recode, (M) Radiation recode, (N) Tumor size. Abbreviations: OS: overall survival.
