# Supplementary material for: Development and validation of a prognostic model to predict the prognosis of patients with colorectal gastrointestinal stromal tumor: A large international population-based cohort study
Source: Front Oncol. 2022 Nov 2;12:1004662. doi: 10.3389/fonc.2022.1004662 (PMC9666406; doi:10.3389/fonc.2022.1004662)
Supplement: Supplementary file 3 [file Table_3.docx]

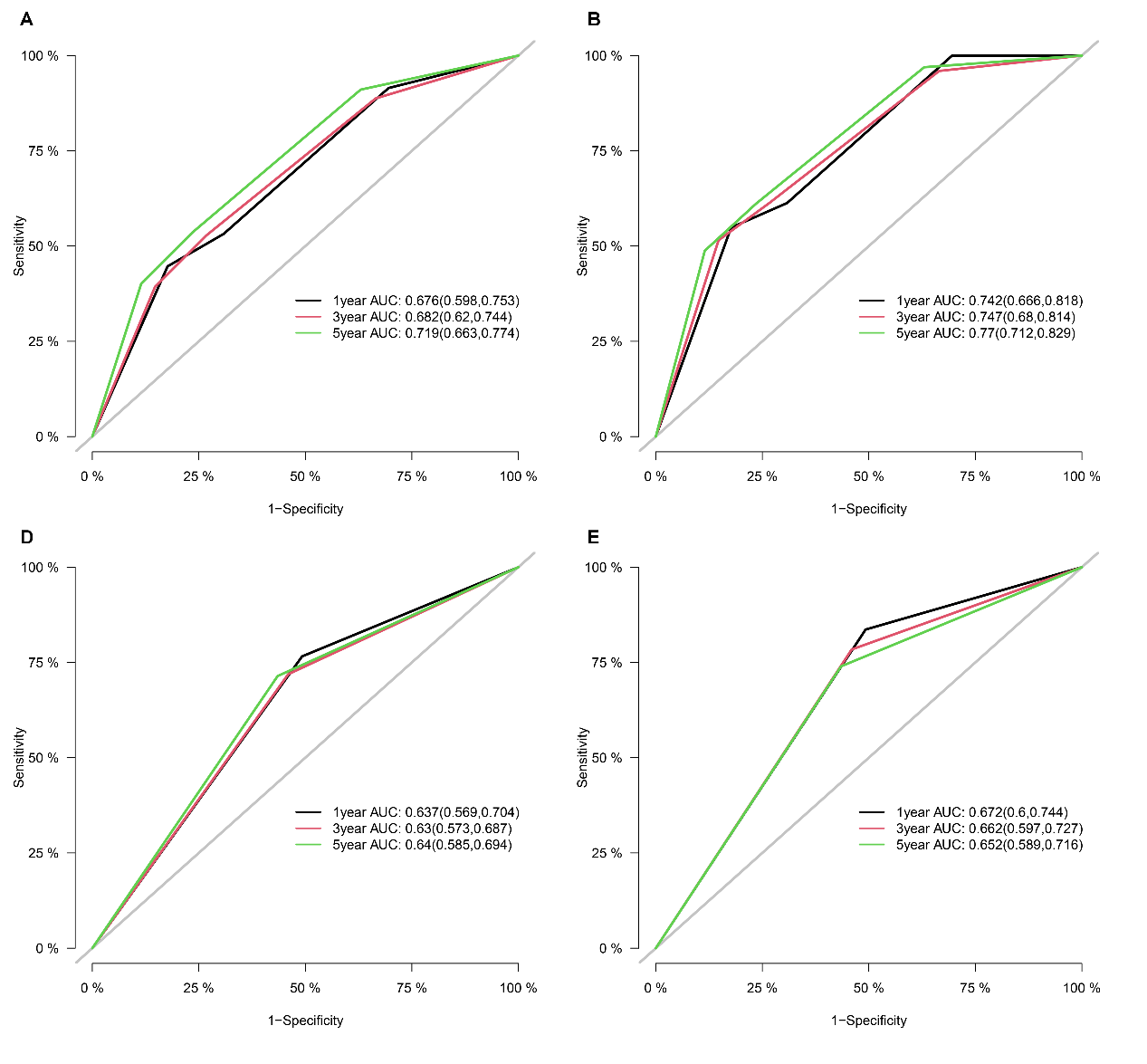


**Supplementary Figure 3** ROCs curve for the TNM staging system and histological grade in predicting prognosis in patients with Colorectal GISTs. (A) ROC of 1-, 3- and 5-year OS of TNM staging system; (B) ROC of 1-, 3- and 5-year CSS of TNM staging system; (C) ROC of 1-, 3- and 5-year OS of histological grade, (D) ROC of 1-, 3- and 5-year CSS of histological grade. Abbreviations: ROC: receiver operating characteristic; OS: overall survival; CSS: cancer-specific survival; Colorectal GISTs: Colorectal gastrointestinal stromal tumors.
